# Supplementary material for: Using Extracellular miRNA Signatures to Identify Patients with LRRK2-Related Parkinson’s Disease
Source: J Parkinsons Dis. 2024 Jul 23;14(5):977–91. doi: 10.3233/JPD-230408 (PMC11307038; doi:10.3233/JPD-230408)
Supplement: Supplementary Material [file jpd-14-jpd230408-s001.pdf]

Supplementary Material

Using Extracellular miRNA Signatures to Identify Patients with LRRK2-Related Parkinson's Disease

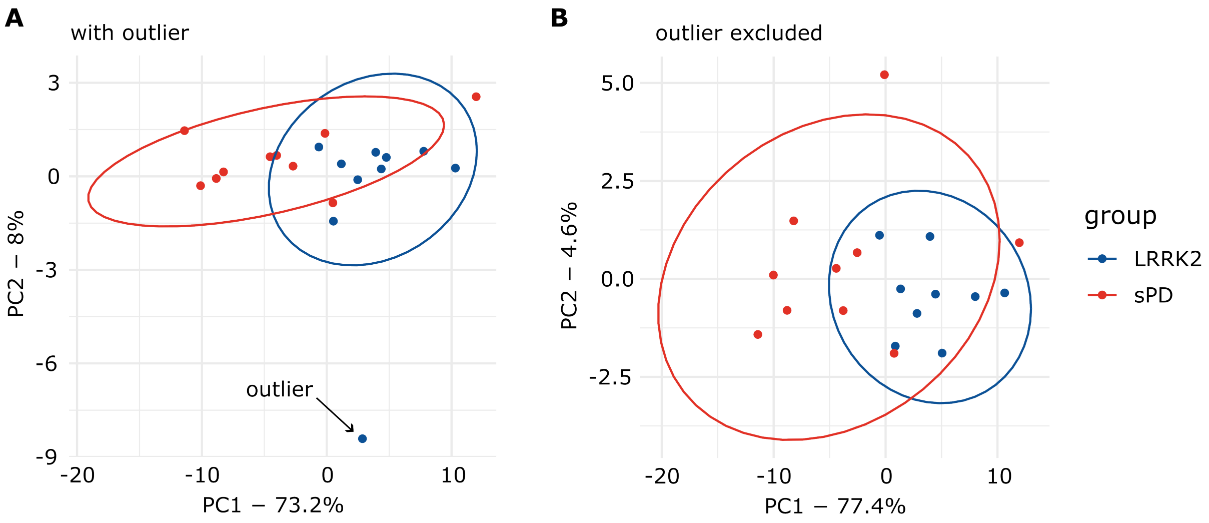

Supplementary Figure 1. Identification of an outlier via PCA. A) The PCA graph on the left displays data points from all sPD patients and $LRRK2_{MC}$. One of the asymptomatic $LRRK2_{MC}$ clearly behaved as an outlier and was therefore excluded from further analysis. B) PCA was repeated with the outlier excluded.

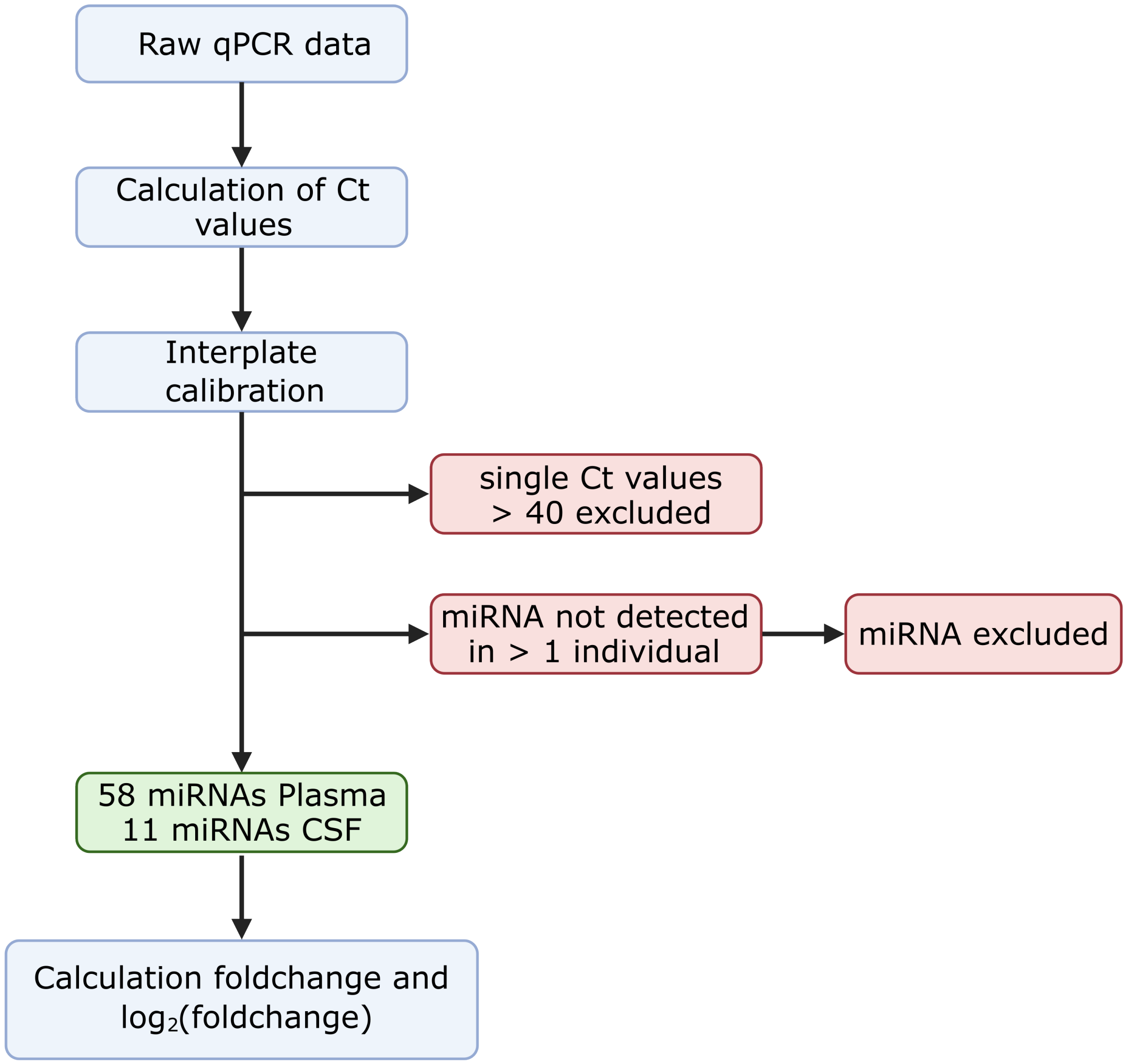

Supplementary Figure 2. Processing of Raw data. Overview of data processing and selection process. Created with BioRender.com.

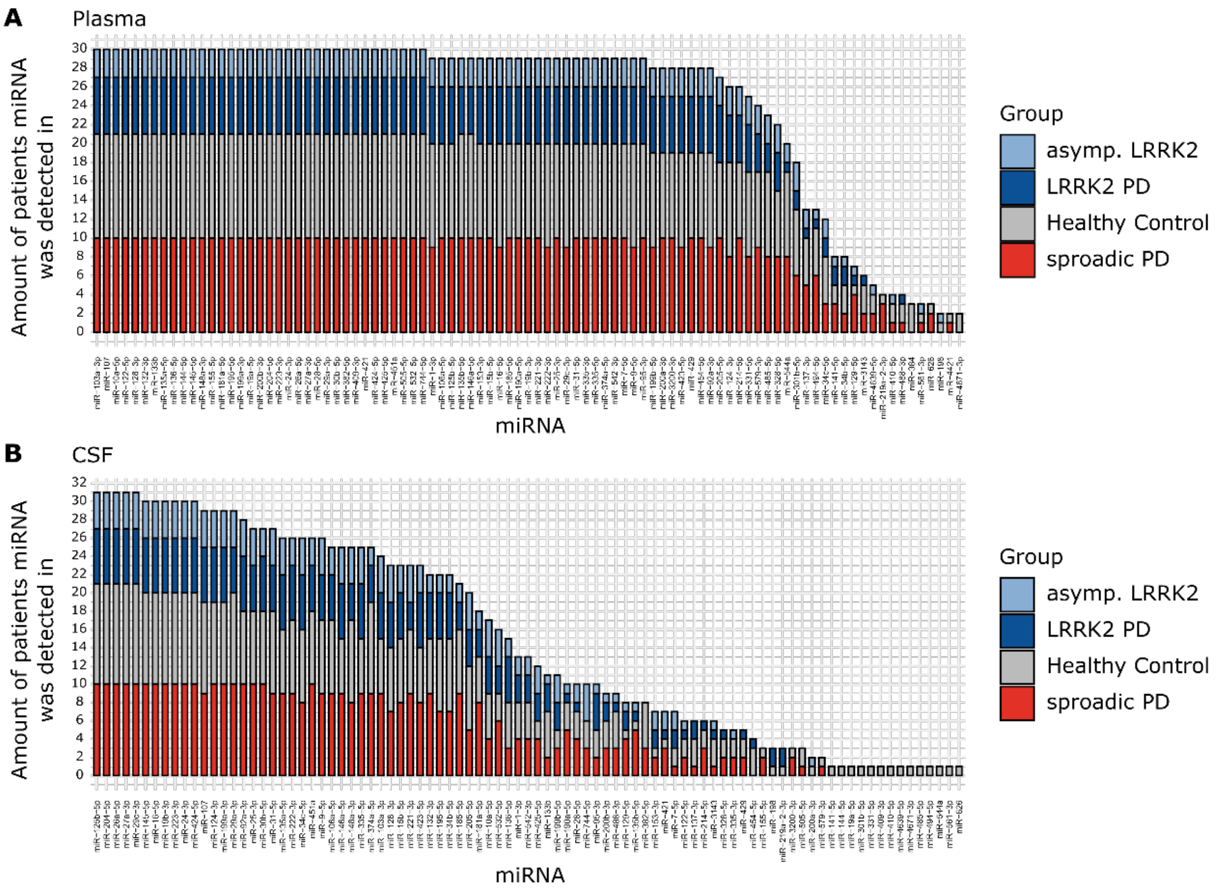

Supplementary Figure 3. Group-wise Expression Distribution of miRNAs. A) The histograms show the number of individuals any given miRNA (x-axis) was found to be expressed in, either in plasma or B) CSF. Colors indicate group affiliation.

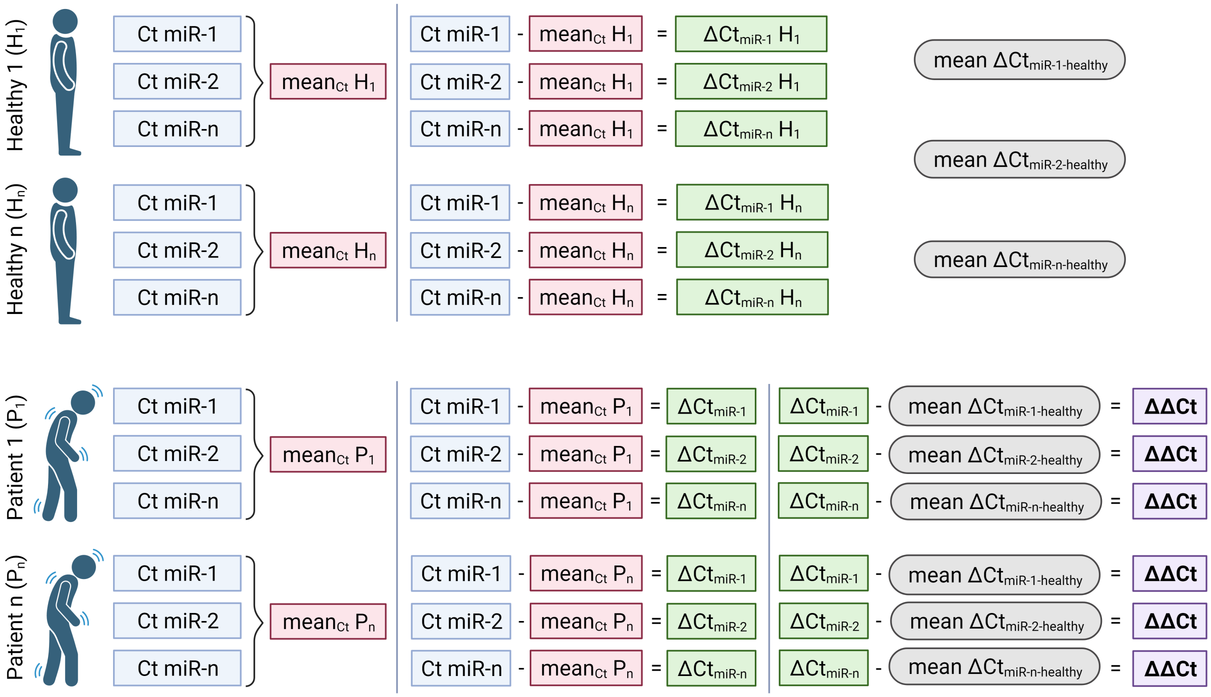

Supplementary Figure 4. Calculation of ΔΔCt values. Overview of calculation of ΔΔCt values. Created with BioRender.com.

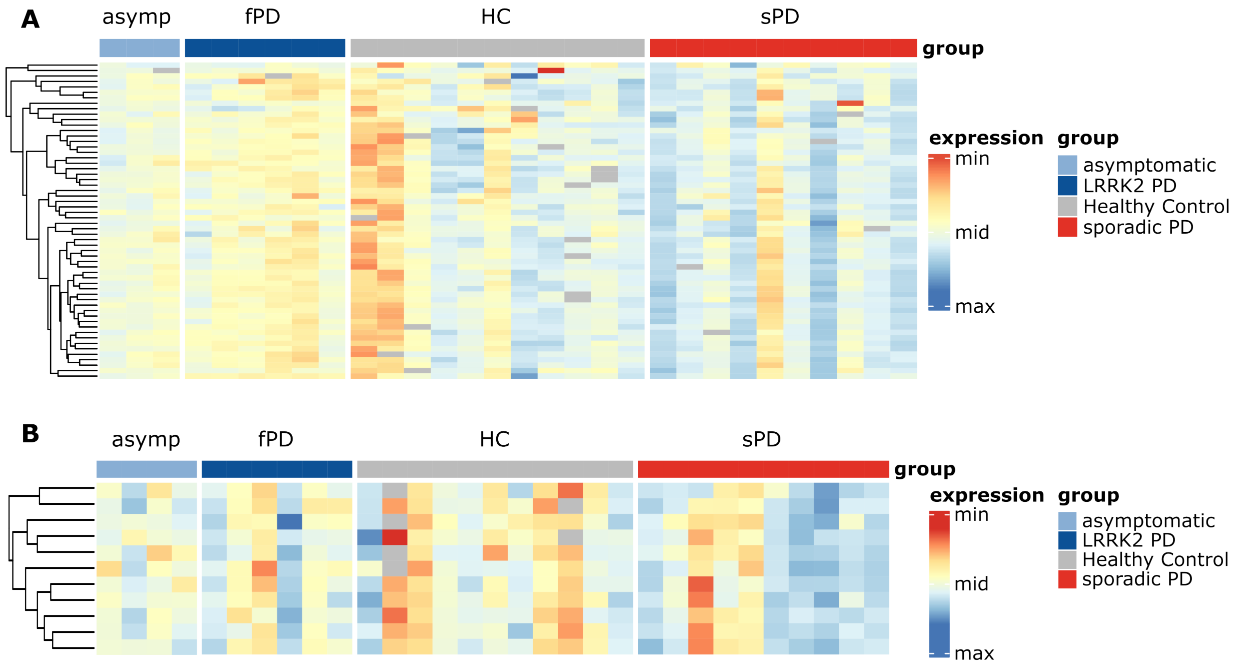

Supplementary Figure 5. Heatmaps based on Ct values. A) Heatmaps for Ct_{plasma} and B) Ct_{CSF}, normalized per row. Rows represent miRNAs, columns represent individuals. Grey cells represent missing values.

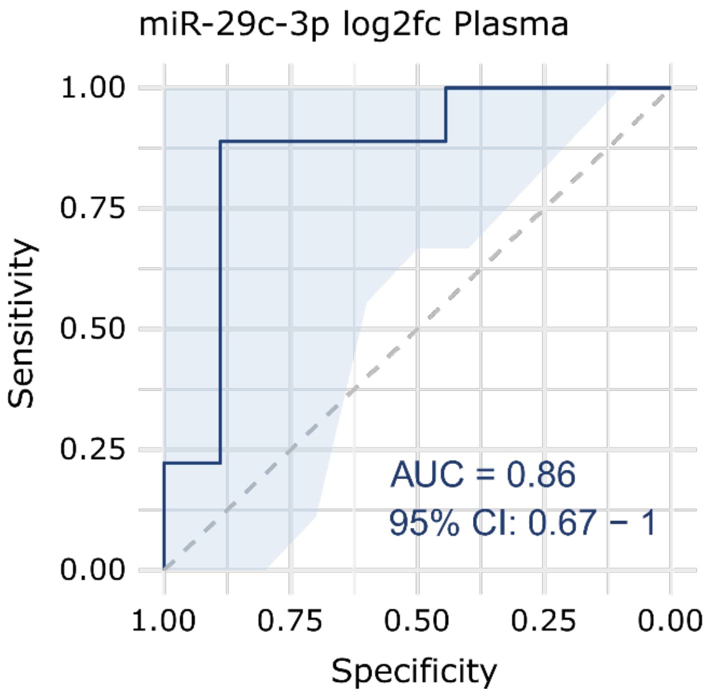

Supplementary Figure 6. ROC curve for miR-29c-3p. ROC curve (dark blue line) with 95% CI (light blue area) displaying the sensitivity and specificity of a classification in $LRRK2_{MC}$ and sPD based on plasma log2fc values of miR-29c-3p.

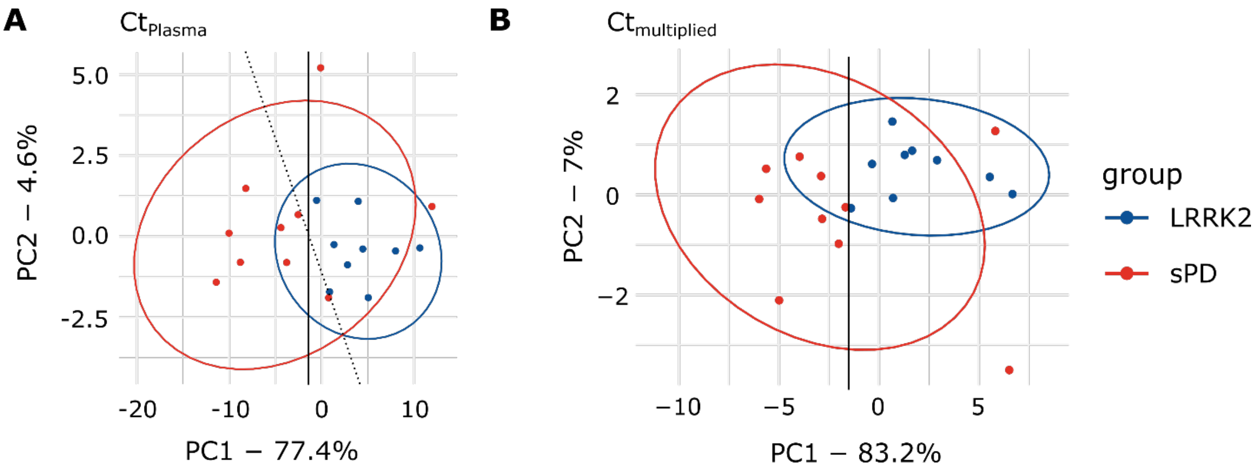

Supplementary Figure 7. PCA graphs including lines for evaluation of discrimination based on sensitivity and specificity. A) Ct_{plasma}. PC1 (solid line) achieves a sensitivity of 100% (9/9) and a specificity of 70% (7/10). PC1 and PC2 combined (dotted line) reach the same sensitivity with a specificity of 80% (8/10). B) $Ct_{multiplied}$. PC1 achieves separation with sensitivity of 100% (9/9) and specificity of 80% (8/10).

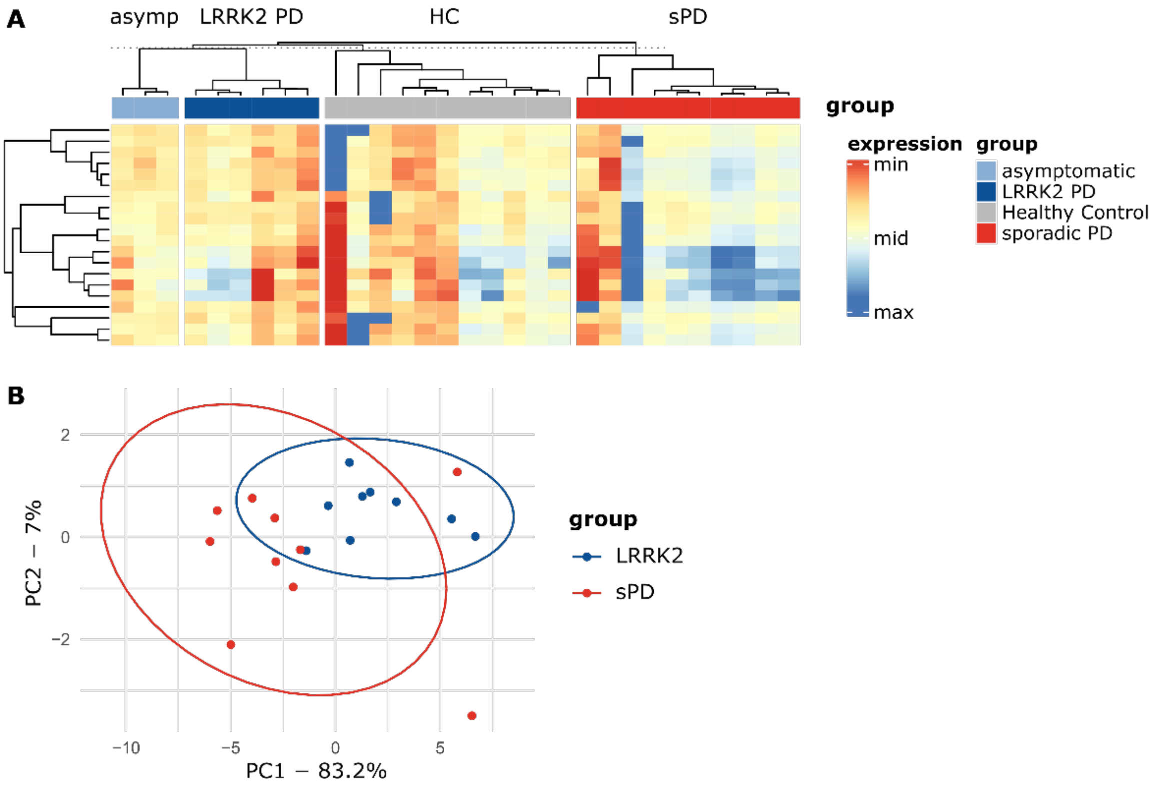

Supplementary Figure 8. Heatmap and PCA resulting from multiplied variables. A) $Ct_{multiplied}$ are plotted as a heatmap for all groups. Again, rows display miRNAs, while columns display individuals. B) When performing PCA with $Ct_{multiplied}$, group separation was observable.

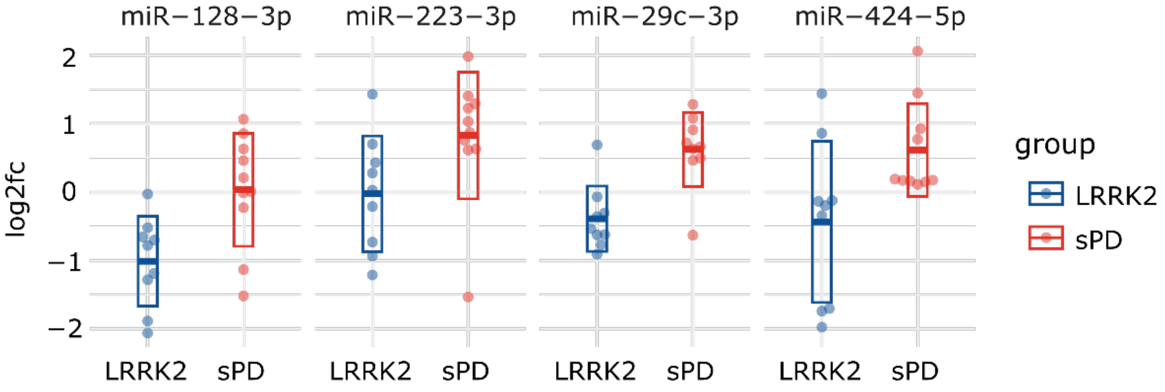

Supplementary Figure 9. Scatterplots of log2fc values for the four discussed miRNAs. Thick line indicates mean while the box indicates the standard deviation.

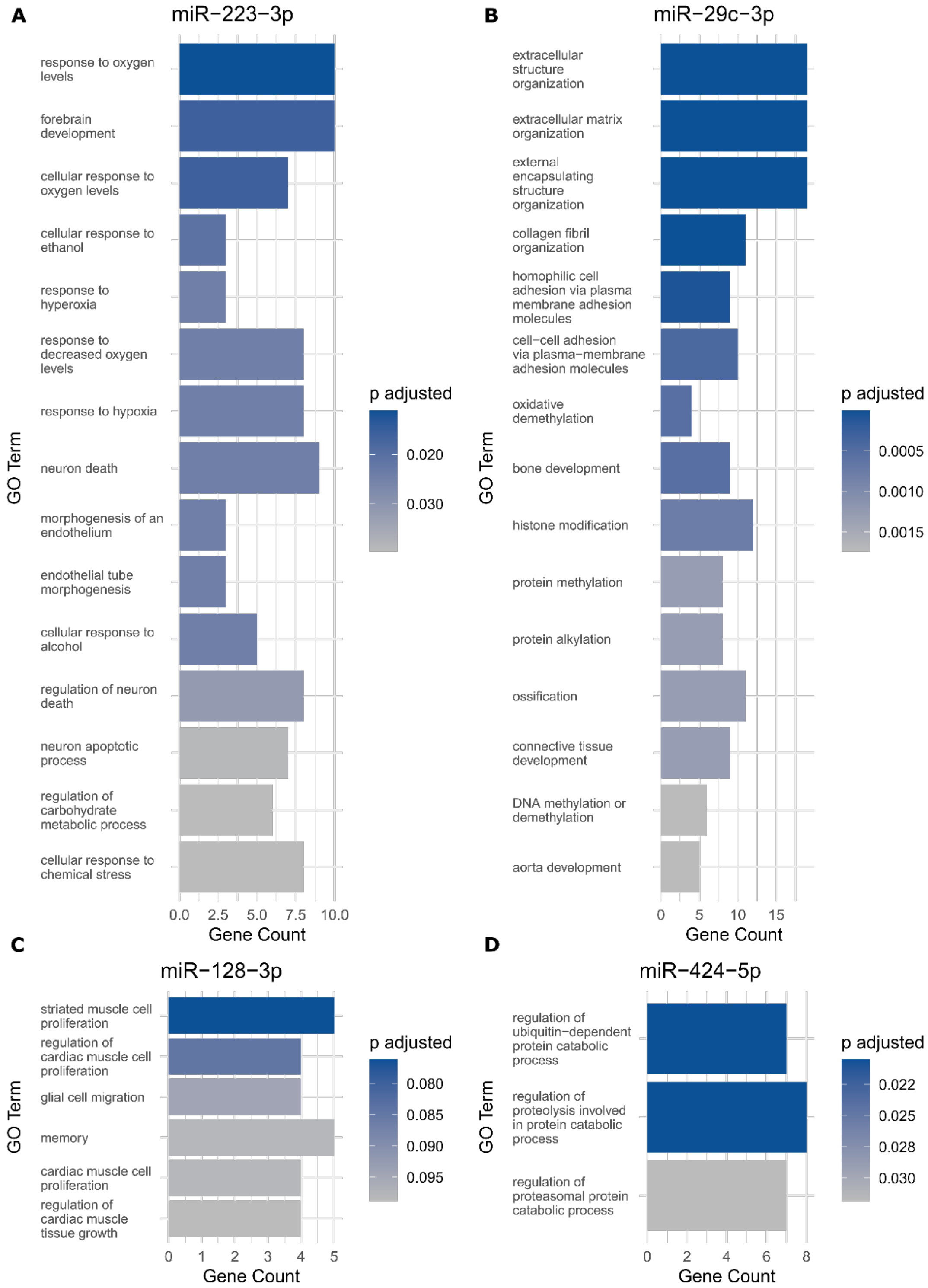

Supplementary Figure 10. GO analysis of identified miRNAs. A) miR-223-3p. 25 significant annotations were identified and sorted by adjusted p-value. Top 15 annotations are displayed. B) miR-29c-3p. 60 significant annotations were identified, top 15 annotations are displayed. C) miR-128-3p. There were no annotations with an adjusted p-value < 0.05. 6 annotations with an adjusted p-value < 0.01 are displayed. D) miR-424-5p. 3 Annotations with an adjusted p-value < 0.05 were identified.

Supplementary Table 1. Included miRNAs with literature references.

miRNA	Context	Reference
miR-16-5p	Striatal tissue of LRRK2 KO mice	[36]
miR-1-3p	Exosomal miRNAs in CSF of PD patients	[14]
miR-103a-3p	Striatal tissue of LRRK2 KO mice	[36]
miR-106a-5p	Brain tissue of PD patients	[30]
miR-107	Algorithm based predicted interaction with LRRK2	[37]
	Plasma of AD and PD patients	[38]
miR-10a-5p	Exosomal miRNAs in CSF of PD patients	[14]
miR-122-5p	Striatal tissue of LRRK2 KO mice	[36]
miR-124-3p	Plasma of PD patients	[39]
miR-125b-5p	CSF of AD patients	[40]
miR-128-3p	Brain tissue of mouse models of PD	[41]
miR-129-5p	Algorithm based predicted interaction with LRRK2	[37]
miR-132-3p	Brain tissue of PD patients	[30]
miR-133b	Plasma of PD patients	[42]
miR-135a-5p	Brain tissue of PD patients	[30]
miR-135b-5p	Brain tissue of PD patients	[43]
miR-136-5p	Algorithm based predicted interaction with LRRK2	[37]
miR-137-3p	Plasma of PD patients	[39]
miR-141-5p	Algorithm based predicted interaction with LRRK2	[37]
miR-144-5p	CSF of PD patients	[44]
	Algorithm based predicted interaction with LRRK2	[37]
miR-145-5p	Brain tissue of PD patients	[30]
miR-146a-5p	Serum of LRRK2 PD patients	[45]
miR-148a-3p	Brain tissue of PD patients	[30]
miR-153-3p	Exosomal miRNAs in CSF of PD patients	[14]
miR-155-5p	Serum of LRRK2 PD patients	[45]
miR-15b-5p	Serum of PD patients	[46]
miR-181a-5p	Serum of PD patients	[46]
	Algorithm based predicted interaction with LRRK2	[37]
miR-185-5p	Serum of PD patients	[46]
	Algorithm based predicted interaction with LRRK2	[37]
miR-190a-5p	Brain tissue of PD patients	[34]
miR-195-5p	Serum of PD patients	[46]
miR-198	Brain tissue of PD patients	[43]
miR-199a-3p	Brain tissue of PD patients	[34]
miR-199b-5p	Brain tissue of PD patients	[47]
miR-19a-5p	Serum of LRRK2 PD patients	[26]
	Algorithm based predicted interaction with LRRK2	[37]
miR-19b-3p	Serum of PD patients	[26]
	Algorithm based predicted interaction with LRRK2	[37]

	Exosomal miRNAs in CSF of PD patients	[14]
miR-200a-3p	CSF of PD patients	[44]
	Algorithm based predicted interaction with LRRK2	[37]
miR-200b-3p	Algorithm based predicted interaction with LRRK2	[37]
miR-204-5p	Brain tissue of PD patients	[48]
miR-205-5p	CSF of PD patients	[49]
	Algorithm based predicted interaction with LRRK2	[37]
miR-214-5p	Serum of PD patients	[50]
miR-219a-2-3p	Brain tissue of PD patients	[48]
miR-221-3p	Plasma of PD patients	[42]
miR-222-3p	Plasma of PD patients	[51]
miR-223-3p	Brain tissue of PD patients	[30]
miR-24-3p	CSF of PD patients	[49]
miR-25-3p	Algorithm based predicted interaction with LRRK2	[37]
miR-26a-5p	Brain tissue of PD patients	[30]
miR-27a-3p	CSF of PD patients	[52]
miR-28-5p	Brain tissue of PD patients	[30]
miR-29a-3p	Serum of LRRK2 PD patients	[26]
miR-29c-3p	Serum of LRRK2 PD patients	[26]
miR-301b-5p	Algorithm based predicted interaction with LRRK2	[37]
miR-30b-5p	Serum of PD patients	[53]
miR-31-5p	Serum of PD advanced PD patients	[50]
miR-3143	Whole blood of PD patients	[54]
miR-3200-3p	Brain tissue of PD patients	[48]
miR-328-5p	Algorithm based predicted interaction with LRRK2	[37]
miR-331-5p	Exosomal miRNAs in Plasma of PD patients	[55]
miR-335-3p	Serum of LRRK2 PD patients	[45]
miR-335-5p	Serum of LRRK2 PD patients	[45]
miR-34b-5p	Brain tissue of PD patients	[56]
miR-34c-5p	Brain tissue of PD patients	[56]
miR-374a-5p	Brain tissue of PD patients	[30]
miR-382-5p	Brain tissue of PD patients	[48]
miR-384	Algorithm based predicted interaction with LRRK2	[37]
miR-409-3p	Exosomal miRNAs in CSF of PD patients	[14]
miR-410-5p	Algorithm based predicted interaction with LRRK2	[37]
miR-421	Brain tissue of PD patients	[48]
miR-423-5p	CSF of PD patients	[52]
miR-424-5p	Brain tissue of PD patients	[34]
miR-425-5p	Brain tissue of PD patients	[48]
miR-429	Brain tissue of PD patients	[34]
	Algorithm based predicted interaction with LRRK2	[37]
miR-4421	Brain tissue of PD patients	[48]
miR-451a	Brain tissue of PD patients	[34]

miR-454-5p	Algorithm based predicted interaction with LRRK2	[37]
miR-4639-5p	Plasma of PD patients	[57]
miR-4671-3p	Whole blood of PD patients	[54]
miR-485-5p	Serum of PD patients	[58]
miR-488-3p	Brain tissue of PD patients	[47]
miR-494-5p	Serum of PD patients	[59]
miR-505-5p	Exosomal miRNAs in Plasma of PD patients	[55]
miR-532-5p	Brain tissue of PD patients	[30]
miR-542-3p	CSF of PD patients	[44]
miR-544a	Brain tissue of PD patients	[47]
miR-561-3p	Whole blood of PD patients	[54]
miR-579-3p	Whole blood of PD patients	[54]
miR-626	Plasma of PD patients	[51]
miR-7-5p	Serum of PD patients	[60]
miR-744-5p	Brain tissue of PD patients	[30]
miR-92a-3p	Brain tissue of PD patients	[30]
miR-95-3p	Brain tissue of PD patients	[48]
miR-9-5p	Serum of PD patients	[61]

AD, Alzheimer's disease; PD, Parkinson's disease

Supplementary Table 2. Melting points of miRNAs detected in plasma. Only miRNAs which were selected for further analysis steps are displayed. Low standard deviations indicate constant amplification of the target.

Target	Mean Melting Point [°C]	Standard deviation [°C]
hsa-miR-103a-3p	69.71	0.31
hsa-miR-106a-5p	71.71	0.34
hsa-miR-107	69.73	0.34
hsa-miR-10a-5p	71.77	0.66
hsa-miR-122-5p	70.59	0.31
hsa-miR-125b-5p	71.37	0.38
hsa-miR-128-3p	69.75	0.11
hsa-miR-132-3p	71.20	0.16
hsa-miR-133b	71.54	0.21
hsa-miR-135a-5p	70.62	0.13
hsa-miR-135b-5p	69.36	0.32
hsa-miR-136-5p	70.30	0.13
hsa-miR-1-3p	70.65	0.13
hsa-miR-144-5p	71.39	0.18
hsa-miR-145-5p	69.09	0.32
hsa-miR-146a-5p	70.36	0.17
hsa-miR-148a-3p	69.78	0.27
hsa-miR-153-3p	69.92	0.22
hsa-miR-155-5p	69.63	0.19
hsa-miR-15b-5p	69.34	0.25
hsa-miR-16-5p	70.60	0.18
hsa-miR-181a-5p	70.26	0.17
hsa-miR-185-5p	70.65	0.16
hsa-miR-190a-5p	69.29	0.26
hsa-miR-195-5p	69.31	0.28
hsa-miR-199a-3p	69.94	0.13
hsa-miR-19a-5p	70.44	0.15
hsa-miR-19b-3p	69.88	0.15
hsa-miR-200b-3p	71.37	0.13
hsa-miR-204-5p	69.22	0.15
hsa-miR-221-3p	69.93	0.51
hsa-miR-222-3p	69.91	0.40
hsa-miR-223-3p	70.84	0.20
hsa-miR-24-3p	74.47	0.18
hsa-miR-25-3p	69.82	0.26
hsa-miR-26a-5p	70.88	0.12
hsa-miR-27a-3p	70.86	0.20
hsa-miR-28-5p	70.30	0.15

hsa-miR-29a-3p	70.19	0.23
hsa-miR-29c-3p	70.40	0.13
hsa-miR-30b-5p	69.20	0.10
hsa-miR-31-5p	69.22	1.82
hsa-miR-335-3p	70.89	0.13
hsa-miR-335-5p	71.43	0.25
hsa-miR-374a-5p	70.21	0.16
hsa-miR-382-5p	71.78	0.26
hsa-miR-409-3p	70.32	0.26
hsa-miR-421	73.12	0.29
hsa-miR-423-5p	70.77	0.29
hsa-miR-424-5p	69.30	0.12
hsa-miR-425-5p	70.09	0.36
hsa-miR-451a	70.11	0.27
hsa-miR-505-5p	70.23	0.23
hsa-miR-532-5p	69.43	0.45
hsa-miR-542-3p	70.47	0.13
hsa-miR-744-5p	73.34	0.43
hsa-miR-7-5p	70.12	0.36
hsa-miR-95-3p	69.48	0.38
hsa-miR-9-5p	73.70	0.49

Supplementary Table 3. Melting points of miRNAs detected in CSF. Only miRNAs which were selected for further analysis steps are displayed. Low standard deviations indicate constant amplification of the target.

Target	Mean Melting Point [°C]	Standard deviation [°C]
hsa-miR-16-5p	70.84	0.47
hsa-miR-125b-5p	71.32	0.43
hsa-miR-145-5p	69.12	0.33
hsa-miR-19b-3p	69.75	0.27
hsa-miR-204-5p	69.25	0.15
hsa-miR-223-3p	70.66	0.38
hsa-miR-24-3p	74.19	1.05
hsa-miR-26a-5p	71.05	0.84
hsa-miR-27a-3p	70.83	0.29
hsa-miR-29c-3p	70.55	0.25
hsa-miR-424-5p	69.42	0.22

Supplementary Table 4. Statistic details from plasma t-tests. Alpha was corrected to 0.0009 by dividing by the number of tests.

t-statistic	p	miRNA
-1.74	0.1019	hsa-miR-1-3p
-2.10	0.0524	hsa-miR-103a-3p
-2.72	0.0146	hsa-miR-106a-5p
-2.50	0.0239	hsa-miR-107
-0.25	0.8073	hsa-miR-10a-5p
2.62	0.0218	hsa-miR-122-5p
0.72	0.4803	hsa-miR-125b-5p
-3.06	0.0071	hsa-miR-128-3p
-1.98	0.0658	hsa-miR-132-3p
-1.39	0.1841	hsa-miR-133b
-2.74	0.0161	hsa-miR-135a-5p
-1.25	0.2296	hsa-miR-135b-5p
-1.89	0.0764	hsa-miR-136-5p
1.37	0.1998	hsa-miR-144-5p
-1.56	0.1384	hsa-miR-145-5p
-1.90	0.0783	hsa-miR-146a-5p
-3.31	0.0042	hsa-miR-148a-3p
-3.58	0.0035	hsa-miR-153-3p
-2.02	0.0597	hsa-miR-155-5p
-1.57	0.1357	hsa-miR-15b-5p
0.08	0.9378	hsa-miR-16-5p
-1.84	0.0851	hsa-miR-181a-5p
-3.00	0.0081	hsa-miR-185-5p
-2.23	0.0431	hsa-miR-190a-5p
-2.60	0.0202	hsa-miR-195-5p
-2.04	0.0588	hsa-miR-199a-3p
-1.02	0.3222	hsa-miR-19a-5p
-1.96	0.0734	hsa-miR-19b-3p
-1.19	0.2502	hsa-miR-200b-3p
-0.94	0.3590	hsa-miR-204-5p
-1.37	0.1908	hsa-miR-221-3p
-2.06	0.0574	hsa-miR-222-3p
-2.10	0.0514	hsa-miR-223-3p
-1.15	0.2666	hsa-miR-24-3p
-0.82	0.4257	hsa-miR-25-3p
-2.02	0.0658	hsa-miR-26a-5p
-1.86	0.0822	hsa-miR-27a-3p
-2.33	0.0358	hsa-miR-28-5p
-1.73	0.1064	hsa-miR-29a-3p

-4.21	**0.0007***	hsa-miR-29c-3p
-2.07	0.0535	hsa-miR-30b-5p
-1.55	0.1505	hsa-miR-31-5p
-1.51	0.1489	hsa-miR-335-3p
-2.24	0.0397	hsa-miR-335-5p
-2.61	0.0186	hsa-miR-374a-5p
-1.21	0.2421	hsa-miR-382-5p
-1.27	0.2233	hsa-miR-409-3p
-1.31	0.2068	hsa-miR-421
-2.35	0.0360	hsa-miR-424-5p
-4.01	0.0010	hsa-miR-425-5p
-0.62	0.5441	hsa-miR-451a
-1.36	0.1945	hsa-miR-505-5p
-2.08	0.0528	hsa-miR-532-5p
0.25	0.8022	hsa-miR-542-3p
-1.26	0.2261	hsa-miR-7-5p
-1.10	0.2890	hsa-miR-744-5p
-2.75	0.0148	hsa-miR-9-5p
1.68	0.1106	hsa-miR-95-3p

Supplementary Table 5. Statistics of correlation matrix. Correlations calculated after Pearson. Only significant miRNA combinations ($p < 0.05$) are displayed.

miRNA Plasma	miRNA CSF	R	R^2	p
miR-16-5p	miR-125-5p	0.39	0.15	0.037
miR-16-5p	miR-145-5p	0.39	0.15	0.042
miR-16-5p	miR-19b-3p	0.40	0.16	0.034
miR-16-5p	miR-223-3p	0.43	0.19	0.021
miR-16-5p	miR-27a-3p	0.46	0.21	0.013
miR-19b-3p	miR-125-5p	0.38	0.15	0.041
miR-19b-3p	miR-19b-3p	0.44	0.20	0.018
miR-19b-3p	miR-223-3p	0.48	0.23	0.010
miR-19b-3p	miR-27a-3p	0.45	0.20	0.014
miR-204-5p	miR-125b-5p	0.37	0.14	0.043
miR-204-5p	miR-19b-3p	0.44	0.19	0.018
miR-204-5p	miR-223-3p	0.40	0.16	0.032
miR-204-5p	miR-27a-3p	0.48	0.23	0.007
miR-204-5p	miR-29c-3p	0.41	0.17	0.023
miR-223-3p	miR-223-3p	0.51	0.26	0.005
miR-26a-5p	miR-223-3p	0.49	0.24	0.007
miR-26a-5p	miR-27a-3p	0.36	0.13	0.032
miR-27a-3p	miR-223-3p	0.50	0.25	0.006
miR-27a-3p	miR-27a-3p	0.39	0.15	0.032
miR-29c-3p	miR-27a-3p	0.38	0.14	0.043

Supplementary Table 6. t-test with $Ct_{multiplied}$ data. p-value threshold was corrected for multiple testing by dividing by the number of tests and set to 0.0025.

miRNA Plasma	miRNA CSF	t-statistic	p
miR-16-5p	miR-125-5p	0.8	0.4483
miR-16-5p	miR-145-5p	1.1	0.3086
miR-16-5p	miR-19b-3p	2.0	0.0599
miR-16-5p	miR-223-3p	2.1	0.0500
miR-16-5p	miR-27a-3p	1.4	0.1898
miR-19b-3p	miR-125-5p	1.5	0.1602
miR-19b-3p	miR-19b-3p	2.8	0.0132
miR-19b-3p	miR-223-3p	2.8	0.0119
miR-19b-3p	miR-27a-3p	2.1	0.0544
miR-204-5p	miR-125b-5p	1.0	0.3343
miR-204-5p	miR-19b-3p	2.1	0.0517
miR-204-5p	miR-223-3p	2.3	0.0324
miR-204-5p	miR-27a-3p	1.5	0.1457
miR-204-5p	miR-29c-3p	1.3	0.2131
miR-223-3p	miR-223-3p	2.9	0.0113
miR-26a-5p	miR-223-3p	2.6	0.0223
miR-26a-5p	miR-27a-3p	2.2	0.0467
miR-27a-3p	miR-223-3p	2.6	0.0204
miR-27a-3p	miR-27a-3p	2.1	0.0532
miR-29c-3p	miR-27a-3p	4.2	**0.0007***
